# Supplementary material for: HIVprotI: an integrated web based platform for prediction and design of HIV proteins inhibitors
Source: J Cheminform. 2018 Mar 9;10:12. doi: 10.1186/s13321-018-0266-y (PMC5845081; doi:10.1186/s13321-018-0266-y)
Supplement: Supplementary file 2 — Additional file 2. Source code of HIVProtI web server. [file 13321_2018_266_MOESM2_ESM.zip › HIVprotI_Source-code/hivprotI/weka-3-6-6/doc/weka/classifiers/rules/part/MakeDecList.html]

MakeDecList


---


|  |  |  |
| --- | --- | --- |
| **Overview** \| **Package** \| **Class** \| **Tree** \| **Deprecated** \| **Index** \| **Help** \| **Weka's home** | |  |
| **PREV CLASS**   **NEXT CLASS** | **FRAMES**    **NO FRAMES**     **All Classes** |  |
| SUMMARY: NESTED \| FIELD \| CONSTR \| METHOD | DETAIL: FIELD \| CONSTR \| METHOD |  |


---


## weka.classifiers.rules.part Class MakeDecList

```
java.lang.Object
  weka.classifiers.rules.part.MakeDecList
```

**All Implemented Interfaces:**: java.io.Serializable, CapabilitiesHandler, RevisionHandler

---

``` public class MakeDecList extends java.lang.Object implements java.io.Serializable, CapabilitiesHandler, RevisionHandler ```

Class for handling a decision list.

**Version:**
:   $Revision: 5529 $

**Author:**
:   Eibe Frank (eibe@cs.waikato.ac.nz)

**See Also:**: Serialized Form

---

**Constructor Summary**

`MakeDecList(ModelSelection toSelectLocModel,
double cf,
int minNum)`
  
          Constructor for dec list pruned using C4.5 pruning.

`MakeDecList(ModelSelection toSelectLocModel,
int minNum)`
  
          Constructor for unpruned dec list.

`MakeDecList(ModelSelection toSelectLocModel,
int num,
int minNum,
int seed)`
  
          Constructor for dec list pruned using hold-out pruning.


| **Method Summary** | |
| --- | --- |
| `void` | `buildClassifier(Instances data)`             Builds dec list. |
| `double` | `classifyInstance(Instance instance)`             Classifies an instance. |
| `double[]` | `distributionForInstance(Instance instance)`             Returns the class distribution for an instance. |
| `Capabilities` | `getCapabilities()`             Returns default capabilities of the classifier. |
| `java.lang.String` | `getRevision()`             Returns the revision string. |
| `int` | `numRules()`             Outputs the number of rules in the classifier. |
| `java.lang.String` | `toString()`             Outputs the classifier into a string. |

**Methods inherited from class java.lang.Object**

`equals, getClass, hashCode, notify, notifyAll, wait, wait, wait`

**Constructor Detail**

### MakeDecList

```
public MakeDecList(ModelSelection toSelectLocModel,
                   int minNum)
```

:   Constructor for unpruned dec list.

---


### MakeDecList

```
public MakeDecList(ModelSelection toSelectLocModel,
                   double cf,
                   int minNum)
```

:   Constructor for dec list pruned using C4.5 pruning.

---


### MakeDecList

```
public MakeDecList(ModelSelection toSelectLocModel,
                   int num,
                   int minNum,
                   int seed)
```

:   Constructor for dec list pruned using hold-out pruning.


**Method Detail**

### getCapabilities

```
public Capabilities getCapabilities()
```

:   Returns default capabilities of the classifier.

    :   **Specified by:**: `getCapabilities` in interface `CapabilitiesHandler`
    :   **Returns:**: the capabilities of this classifier **See Also:**: `Capabilities`

---


### buildClassifier

```
public void buildClassifier(Instances data)
                     throws java.lang.Exception
```

:   Builds dec list.

    :   **Throws:**: `java.lang.Exception` - if dec list can't be built successfully

---


### toString

```
public java.lang.String toString()
```

:   Outputs the classifier into a string.

    :   **Overrides:**: `toString` in class `java.lang.Object`

---


### classifyInstance

```
public double classifyInstance(Instance instance)
                        throws java.lang.Exception
```

:   Classifies an instance.

    :   **Throws:**: `java.lang.Exception` - if instance can't be classified

---


### distributionForInstance

```
public double[] distributionForInstance(Instance instance)
                                 throws java.lang.Exception
```

:   Returns the class distribution for an instance.

    :   **Throws:**: `java.lang.Exception` - if distribution can't be computed

---


### numRules

```
public int numRules()
```

:   Outputs the number of rules in the classifier.

---


### getRevision

```
public java.lang.String getRevision()
```

:   Returns the revision string.

    :   **Specified by:**: `getRevision` in interface `RevisionHandler`
    :   **Returns:**: the revision


---


|  |  |  |
| --- | --- | --- |
| **Overview** \| **Package** \| **Class** \| **Tree** \| **Deprecated** \| **Index** \| **Help** \| **Weka's home** | |  |
| **PREV CLASS**   **NEXT CLASS** | **FRAMES**    **NO FRAMES**     **All Classes** |  |
| SUMMARY: NESTED \| FIELD \| CONSTR \| METHOD | DETAIL: FIELD \| CONSTR \| METHOD |  |


---
